# Effects of Gingerbread Cookie Enrichment with Native and Supercritical CO_2_-Defatted Burdock Seeds

**DOI:** 10.3390/foods15071115

**Published:** 2026-03-24

**Authors:** Katarina Šavikin, Jelena Živković, Dejan Pljevljakušić, Olivera Šimurina, Bojana Filipčev, Vesna Vujasinović, Elizabeta Dimitrieska Stojkovikj, Sanja Kostadinović Veličkovska

**Affiliations:** 1 Institute for Medicinal Plant Research “Dr. Josif Pančić”, Tadeuša Košćuška 1, 11000 Belgrade, Serbia; ksavikin@mocbilja.rs (K.Š.); jzivkovic@mocbilja.rs (J.Ž.); dpljevljakusic@mocbilja.rs (D.P.); 2 Institute of Food Technology in Novi Sad, University of Novi Sad, Bulevar Cara Lazara 1, 21000 Novi Sad, Serbia; olivera.simurina@fins.uns.ac.rs (O.Š.); bojana.filipcev@fins.uns.ac.rs (B.F.); 3 Faculty of Sciences, University of Novi Sad, Trg Dositeja Obradovića 3, 21000 Novi Sad, Serbia; vesna.vujasinovic@dgt.uns.ac.rs; 4 Faculty of Veterinary Medicine-Skopje, Ss. Cyril and Methodius University, 1000 Skopje, North Macedonia; edimitrieska@fvm.ukim.edu.mk; 5 Faculty of Agriculture, Goce Delchev University, 2000 Shtip, North Macedonia

**Keywords:** burdock seeds, cookies, lignans, phenolic acids, sensorial analysis

## Abstract

Burdock (*Arctium lappa* L., Asteraceae) seeds, a rich source of dietary fibre, proteins, essential and fatty acids, also contain high levels of polyphenols and lignans, especially arctigenin and arctiin. This study investigated the incorporation of native and supercritical CO_2_-defatted burdock seed flour into gingerbread cookies formulated with sweetener xylitol compared to burdock seeds’ free sugar-based and xylitol-based cookies as a control. Arctiin was the dominant lignan in both native and defatted seed flours (68.30 and 75.16 mg/g, respectively), while isochlorogenic acid was the most abundant phenolic acid (7.01 and 7.86 mg/g, respectively). Among enriched formulations, xylitol cookies with defatted burdock seed flour exhibited the highest soluble dietary fibre content (0.29 g/100 g) and reduced hardness, comparable to the xylitol control. All samples achieved “good” sensory quality (18.33–19.65 points), with no significant differences among formulations (*p* > 0.05). Storage studies (60 days) under varying temperature and light conditions revealed a significant decline in sensory quality only for sucrose-based control cookies stored at 40 °C. The concentrations of major phenolic compounds remained stable under all storage conditions. These results demonstrate the technological and nutritional potential of defatted burdock seed flour as a functional ingredient in bakery products.

## 1. Introduction

Burdock (*Arctium lappa* L., Asteraceae) is a well-known plant species with a long history of use in both human nutrition and herbal medicine, particularly in East Asia. In Asian dietary traditions, *A. lappa* root is regularly consumed following simple domestic processing, and its extracts have been widely reported to possess multiple pharmacological properties, ranging from metabolic regulation to neuroprotective activity [1,2]. *A. lappa* root (*Bardanae radix*) is covered by official monographs of the European Medicines Agency (EMA), the Physicians’ Desk Reference (PDR), and the European Scientific Cooperative on Phytotherapy (ESCOP) [3,4,5]. In contrast, burdock seeds are currently officially recognized only in the pharmacopeias of China and Japan [6,7].

Burdock seeds are rich in dietary fibre, proteins, essential fatty acids, and polyphenols. Compared with the roots, the seeds contain higher levels of arctigenin and its glycoside arctiin, lignans associated with a wide range of biological activities, including antihyperglycemic, antidiabetic, skin anti-ageing, and antitumor effects [8,9]. In our previous study, arctiin and arctigenin were shown to be strong α-amylase inhibitors in a concentration-dependent manner. Moreover, the antidiabetic activity of burdock seed extracts was also demonstrated in vivo using eight-week-old male C57BL/6J mice [8].

In addition to their pharmacological relevance, lignans and phenolic compounds are well recognized for their antioxidant activity. These compounds may contribute to reducing oxidative processes in food systems and provide additional nutritional value when incorporated into cereal-based products. Therefore, their presence may support the development of functional bakery products with potential health-promoting properties, particularly in formulations aimed at improving dietary quality or supporting glycemic control [10,11].

Bakery products, such as bread, cookies, and related items, are widely accepted and consumed worldwide due to their pleasant and sweet taste [12]. As consumers are increasingly interested in functional and health-oriented foods, bakery products represent convenient, ready-to-eat matrices for the delivery of health benefits [13,14]. Previous studies have reported improvements in the nutritional value of cookies in which wheat flour was partially replaced with various types of herbal powders [15]. The chemical composition of burdock seeds makes them an attractive ingredient for incorporation into different bakery products. Lee [15] reported the incorporation of burdock seed powder into cookies and noted that sensory analysis indicated an unpleasant taste when the level of seed addition exceeded 6%. Incorporation of plant materials rich in polyphenols and lignans into bakery formulations has also been associated with increased antioxidant capacity and improved functional properties of the final products [10,11]. Previous studies have shown that the addition of burdock powder to wheat cookies (10–30%) can significantly enhance antioxidant activity due to the presence of phenolic compounds while maintaining acceptable physicochemical and sensory properties, particularly at the 10% substitution level [16].

The aim of this study was to evaluate the effects of incorporating raw and supercritical CO_2_ defatted burdock seed flour into gingerbread cookies formulated with sweetener xylitol compared to sugar-based and xylitol-based cookies as controls. We hypothesized that defatted burdock seed flour would improve the nutritional and functional properties of cookies while maintaining acceptable product quality, owing to its reduced fat content and increased relative abundance of bioactive lignans. Additionally, we hypothesized that defatting would enhance oxidative stability and sensory acceptability compared with native seed flour, supporting the valorization of burdock seed as a sustainable functional ingredient in bakery products. Although previous studies have investigated the incorporation of burdock powder or burdock root flour into bakery products such as cookies, the present study focuses on burdock seed flour, particularly after supercritical CO_2_ defatting, which has not been widely explored in bakery formulations. The study evaluates its impact on lignan content, nutritional quality, technological properties, and the storage stability of cookies. Moreover, the cookies were formulated using xylitol as a sweetener, enabling the development of a reduced-sugar product with potential benefits for glycemic control. Therefore, this study provides new insights into the potential application of defatted burdock seed flour as a functional ingredient in reduced-sugar bakery products.

## 2. Materials and Methods

### 2.1. Plant Material

Mature achenes of burdock were collected at full physiological maturity from a fallow field located near Pančevo, South Banat, Serbia (44°52′33.28″ N; 20°41′45.03″ E) in October 2024. The harvested material was processed by sieving to remove debris, and empty achenes were separated by air classification. The resulting batch of clean, fully developed seeds was ground to a fine powder using a laboratory mill and stored in vacuum-sealed containers at 4 °C until further analyses.

A portion of the seed powder (100 g) was defatted by supercritical CO_2_ extraction using a Superex SC 1000 supercritical fluid extractor (Superex, Konya, Turkey). The extraction parameters were selected based on literature data describing supercritical CO_2_ extraction of plant matrices containing lipophilic compounds and lignans [17,18]. The ground seeds were loaded into the extraction vessel, and the process was performed under the following conditions: extractor temperature 60 °C; pre-heater temperature 60 °C; separator 1 and separator 2 temperatures 60 °C; extraction time 150 min; operating pressure 300 bar; CO_2_ pump flow rate 100 mL min^−1^; and co-solvent (ethanol) pump flow rate 2 mL min^−1^. Under these conditions, 11.7 percent of oil was removed from the initial seed material. To determine the total oil content of the plant material used in this study, a control Soxhlet extraction with hexane (8 h) was performed, yielding 12.1% oil from the burdock seeds.

### 2.2. Extract Preparation for HPLC Analysis

Native and defatted ground burdock seeds (1 g) were extracted with methanol (20 mL) for 30 min at room temperature using an ultrasonic bath (Sonorex smart ST, Bandelin electronic, Berlin, Germany). Gingerbread samples were treated under analogous conditions: 5 g of homogenized sample were extracted with 25 mL of methanol for 30 min at room temperature in the same ultrasonic bath. After extraction, all samples were filtered and analyzed on the same day.

### 2.3. HPLC Analysis

Analyses of the extracts were performed using an Agilent 1260 RR HPLC system equipped with a diode array detector (190–550 nm) (Agilent, Waldbronn, Germany). Separation was achieved on a reversed-phase Zorbax SB C18 analytical column (150 mm × 4.6 mm i.d., 5 μm particle size). The mobile phase consisted of an aqueous solution of 1% (*v*/*v*) orthophosphoric acid (phase A) and acetonitrile (phase B), using gradient elution as follows: 0–5 min, 98–90% A; 5–15 min, 90% A; 15–20 min, 90–85% A; 20–25 min, 85–70% A; 25–30 min, 70–40% A; and 30–31 min, 40–0% A, followed by a post run time of 1 min. Detection was carried out at 260, 280, 320, and 360 nm. The flow rate was 1 mL min^−1^, the injection volume was 10 μL, and the column temperature was maintained at 25 °C. Individual compounds were identified by comparing their retention times and UV spectra with those of authentic reference standards, and quantified using external calibration curves. All analyses were performed in triplicate, and results were expressed as milligrams per gram of dry weight (mg g^−1^ dw).

The HPLC method used for the identification and quantification of lignans was fully validated within this study, and the validation parameters are presented in Table S1 (Supplementary Materials).

### 2.4. Gingerbread Cookie Ingredients

The basic raw materials used for gingerbread production were obtained from local producers and suppliers. Wholemeal spelt flour (moisture content 12.13% d.m., ash 1.89% d.m.) and wholemeal rye flour (moisture content 11.56% d.m., ash 1.77% d.m.) were supplied by Univeg (Zabalj, Serbia). Granulated sugar was sourced from Crvenka (Serbia), while meadow honey was obtained from Medomiks (Novi Sad, Serbia). Additional ingredients included vegetable fats composed of palm and sunflower oils in varying proportions (Puratos, Serbia), cocoa powder (Dr. Oetker, Bielefeld, Germany), soy lecithin (Sojaprotein, Bečej, Serbia), sodium bicarbonate (Eti Soda, Turkey), and a commercial gingerbread spice mixture (Kotányi, Wolkersdorf, Austria). Xylitol was supplied by KUK Serbia, a regional distributor of specialty food ingredients. Ground burdock seeds and ground burdock press cake obtained after oil extraction were provided by the Institute for Medicinal Plant Research “Dr. Josif Pančić”, Belgrade, Serbia.

### 2.5. Production of Gingerbread Cookies

Table 1 provides the working codes and descriptions of the gingerbread cookie variants prepared in this study. The ingredient composition of gingernut cookie formulations is given in Table 2. The formulation was designed to be suitable for vegetarians, as well as for individuals who observe religious dietary practices and, during certain periods of the year, follow a fasting diet, i.e., consuming foods that do not contain ingredients of animal origin. Xylitol was used as a sugar substitute based on the findings of research conducted by investigators from Slovakia and Poland [19]. The preparation of biscuits was carried out using a procedure described in detail in Filipčev et al. [20].

### 2.6. Determination of Proximate Composition of Gingerbread Cookies

The chemical composition of the biscuits was determined according to standard AOAC procedures [21]. Moisture (method 926.05), ash (method 930.22), crude protein (method 950.36), fat (method 935.38), reducing sugars expressed as invert sugars prior to hydrolysis (method 975.14), and total dietary fibre (method 958.29) were analyzed. Mineral elements were quantified by atomic absorption spectrophotometry using the AOAC method 984.27 with a Varian Spectra AA 10 instrument (Varian Techtron Pty Limited, Mulgrave, Victoria, Australia). The content of total and available carbohydrates was calculated according to FAO/WHO guidelines using equations:

(1) Total carbohydrates (g/100 g) = 100 − (protein+ fat + water + ash + alcohol)

(2) Available carbohydrates (g/100 g) = 100 − (protein+ fat + water + ash + alcohol + total fibres)

### 2.7. Physical and Texture Analysis

Gingerbread cookie samples were individually evaluated for mass, diameter, height, volume, and texture. Cookie dimensions were measured using a digital calliper. The spread ratio was calculated as the ratio of mean diameter to height, with mean diameter obtained by averaging two measurements taken at perpendicular positions. Cookie volume was determined using a laser scanning volumeter (VolScan 600 Profiler, Stable Micro Systems, Godalming, Surrey, UK).

Textural analysis was performed using a texture analyzer (TA-XTplus, Stable Micro Systems, Godalming, Surrey, UK). Cookie samples were penetrated with a cylindrical probe (2 mm diameter) at a test speed of 0.5 mm s^−1^ over a distance of 22 mm. Hardness was defined as the area under the force–time curve. Fracturability was expressed as the linear distance calculated from the force–time curve, representing the cumulative length of the curve during penetration. Higher linear distance values corresponded to increased force fluctuations and, consequently, greater cookie fracturability, whereas lower values indicated smoother force profiles typical of softer products.

All measurements were conducted in 20 replicates and carried out 2 h after baking.

### 2.8. Colour Measurement

The colour of the gingerbread cookie top crust (L*, a*, b*) was measured before and after baking using a Chroma Meter CR 400 (Konica Minolta, Tokyo, Japan) equipped with a CR A33f attachment. The instrument was calibrated with a white standard plate (CR A43) under D65 illumination and a 10° standard observer angle. Measurements were performed in triplicate. Colour parameters were expressed in the CIELab* colour space, where L* represents lightness on a scale from 0 (black) to 100 (white), a* indicates the green to red axis (negative to positive values), and b* indicates the blue to yellow axis (negative to positive values) [22].

### 2.9. Sensory Analysis

Gingerbread cookie samples were evaluated using a weighted scoring sensory system. Sensory testing was approved by the Ethics Committee of the Faculty of Science, University of Novi Sad, No 0601-81/25-61, date: 25 November 2025, and conducted by a 15-member trained panel experienced in the evaluation of grain-based baked products. Sensory attributes were rated on a five-point scale, where 1 represented the lowest and 5 the highest score for each attribute (Table 3). The score obtained for each quality descriptor was multiplied by its corresponding weighting coefficient to calculate the weighted score. Weighting coefficients were set as follows: 1.0 for aroma and aftertaste, 0.8 for mastication, 0.6 for crumb structure and cookie fracture, and 0.5 for upper surface and shape. Sensory descriptors and weighting coefficients were established by three expert panellists in a preliminary trial. Overall cookie quality was assessed based on the total score and classified as very good (20.1–25.0 points), good (15.1–20.0 points), acceptable (11.1–15.0 points), or not acceptable (<11.0 points). The panellists were not aware of the formulation of the samples presented during evaluation. Samples were coded with randomly generated three-digit numbers and presented to panellists in randomized order according to a balanced design, ensuring that neither sample identity nor serving sequence could bias sensory evaluation.

### 2.10. Storage Stability Study

All cookie samples were subjected to stability testing. The samples were placed in capped glass containers and stored for 60 days under different temperature and light conditions, namely at 4 °C in the dark, at 25 °C under light and dark conditions, and at 40 °C in the dark. All samples were stored in identical capped glass containers to ensure uniform packaging conditions. Although relative humidity was not independently controlled, the use of closed containers ensured comparable and stable microenvironmental conditions throughout the storage period. Product stability under the various storage conditions was evaluated by monitoring the contents of the predominant lignans, arctiin and arctigenin, and phenolic acids, including chlorogenic and isochlorogenic acids, as well as textural and sensory properties.

### 2.11. Statistical Analysis

All chemical analyses of raw materials and gingerbread cookie formulations were performed in three independent replicates, while physical and texture analyses were conducted in 20 replicates. Results are presented in tables and figures as mean values ± standard deviation (SD). Differences among treatments for all evaluated parameters were assessed using one-way ANOVA, followed by Tukey’s HSD post hoc test at a significance level of *p* < 0.05. Statistically significant differences between treatments are indicated by different letters. All statistical analyses and graphical representations were performed using the R software environment (R Core Team), version 4.5.2 (R-CRAN).

## 3. Results and Discussion

### 3.1. HPLC Analysis of Native and Defatted Burdock Seeds

Burdock seeds are rich in bioactive compounds, primarily lignans such as arctigenin, arctiin, lappaol, and matairesinol, as well as phenolic acids including chlorogenic, isochlorogenic, and caffeic acids [8,23]. In the present study, arctiin was the predominant lignan detected in both native and defatted burdock seed samples (Table 4). These findings are consistent with our previous investigation of burdock seeds collected from ten different populations, in which arctiin content ranged from 40.6 to 92.6 mg g^−1^. In contrast, arctigenin, the aglycone form of arctiin, was present at significantly lower levels, ranging from 0.7 to 2.7 mg g^−1^ [8].

Comparable results have been reported by Liu et al. [24], who analyzed burdock seed populations from different regions of China and observed arctiin contents ranging from 26.5 to 73.6 mg g^−1^ and arctigenin contents from 1.4 to 8.8 mg g^−1^.

The significantly lower content of the aglycone arctigenin observed in defatted burdock seeds (Table 4) can be attributed to its higher lipophilicity, which makes it more susceptible to extraction during the supercritical CO_2_ defatting process. Isochlorogenic acid was more abundant than chlorogenic acid, with comparable levels detected in both native and defatted seed samples. Consistent with these findings, Liu et al. [24] reported chlorogenic acid contents ranging from 0.3 to 3.6 mg g^−1^ in burdock seed samples from different origins.

### 3.2. Quality Attributes of Gingerbread Cookies

#### 3.2.1. Proximate Composition

The gingerbread cookie formulations exhibited generally similar proximate compositions (Table 5). Significant differences were observed in protein content, which ranged from 7.56 g/100 g in Xyl-DBS to 9.36 g/100 g in Xyl-C. Among the enriched formulations, Xyl-DBS showed a significantly higher content of soluble dietary fibre (0.29 g/100 g). Total reducing sugars were significantly reduced in formulations in which sucrose was replaced by xylitol, compared with the sucrose-based control cookie (Suc-C). Although the observed increase in soluble fibre (0.29 g/100 g) is modest, it may contribute incrementally to the overall dietary fibre intake when such products are consumed regularly. Given that the recommended dietary fibre intake for adults is approximately 25 g per day [25], even small improvements in fibre content of widely consumed foods such as bakery products may support efforts to increase population-level fibre intake.

In accordance with Regulation (EC) No 1924/2006 on nutrition and health claims made on foods, as transposed into national legislation on nutrition and health claims indicated on food labelling, a food product may be classified as “high in fibre” if it contains at least 6 g of dietary fibre per 100 g of product [26]. All analyzed gingerbread cookie formulations met this criterion and can therefore be classified as nutritionally valuable products, which can be attributed to the formulations presented in Table 3.

All changes in the energy value of the studied cookie variants were minor. The energy value of gingerbread cookies declined only by 1% upon sucrose replacement with xylitol. Cookie enrichment with burdock seed tended to slightly increase the energy value. A negligible drop in energy value of gingerbread made with xylitol as sugar replacer was reported by Ivanišova et al. [19]: 447.9 kcal/100 g vs. 480 kcal/100 g in the control sample made with sucrose.

#### 3.2.2. Mineral Content

According to national regulations on nutrition and health claims, harmonized with EU legislation, a food product may be declared a source of a mineral if it provides at least 15% of the daily reference intake, while a “rich in” claim requires a contribution of at least 30% of the daily reference intake of the respective mineral [27,28].

Based on their mineral composition (Table 6), gingerbread cookies formulated with wholemeal spelt and rye flours, with or without the addition of burdock seed ingredients, contributed meaningfully to the intake of essential minerals. Although the contribution of certain minerals to the dietary reference intake (%DRI) may be considered moderate, these values should be interpreted in the context of the nutritional role of cereal-based fine bakery products in the daily diet. Such products are typically consumed as part of a varied diet rather than as the sole source of minerals; therefore, even a moderate contribution may have practical nutritional relevance when considered cumulatively with other foods consumed throughout the day.

Importantly, several minerals reached levels that allow nutrition claims in accordance with Regulation (EC) No 1924/2006. All formulations qualified as a source of iron (Fe) and magnesium (Mg), providing more than 15% of the daily reference intake per 100 g of product. Cookies enriched with burdock seed or defatted burdock seed additionally qualified as a source of zinc (Zn), while all samples met the criteria for being rich in manganese (Mn) and copper (Cu).

From a practical nutritional perspective, the use of wholemeal spelt and rye flours in combination with burdock seed ingredients contributes to an improved mineral profile compared with conventional refined flour fine bakery products. Overall, the improved mineral profile highlights the potential of these gingerbread cookies as nutritionally valuable whole-grain fine bakery products with an enhanced contribution of micronutrients.

#### 3.2.3. Physical and Textural Properties

The physical and textural characteristics of the gingerbread cookies are presented in Table 7. Cookie spread, defined as the ratio of width to thickness, is a key quality attribute influenced by ingredient composition, flour functionality, sugar type, and particle size, as well as baking conditions and ambient humidity. In the present study, the control gingerbread cookies (Suc-C) exhibited the highest spread value (3.56) and the largest specific volume (1.59 g·mL^−1^). Partial replacement of sucrose with xylitol resulted in a statistically significant reduction in both spread (2.76) and specific volume (1.50 g·mL^−1^) (Xyl-C).

These findings contrast with those reported by Ivanišová et al. [19], who observed maximum cookie width and volume in gingerbread formulations prepared with xylitol. The discrepancy may be attributed to differences in flour type and baking performance between studies. In support of this explanation, Kweon et al. [29] demonstrated that replacing sucrose with xylose in sugar-snap cookie formulations adversely affected flour functionality, leading to minimal increases in cookie width and length and a pronounced increase in height, ultimately resulting in reduced spread. Given that xylitol is derived from xylose, similar structural effects on dough behaviour and cookie geometry may partly account for the results observed in the present study.

Besides cookie spread and volume, sugars contribute to several other key quality attributes of cookies, including hardness, crispness, and colour. Slow sugar crystallization during cooling after baking promotes the development of a firm and brittle texture. According to Lin et al. [30], sucrose acts as a hardening agent as a result of its crystallization behaviour. Consequently, the control gingerbread cookies formulated with sucrose (Suc-C) exhibited the highest hardness (3685.2 g·s) and fracturability (1241.1 g·s), whereas cookies in which sucrose was replaced with xylitol (Xyl-C) were significantly softer (1905.4 g·s) and less fracturable (921.2 g·s).

Compared with the xylitol control (Xyl-C), the addition of burdock seed (Xyl-BS) resulted in a slight increase in hardness (2337.3 g·s) and fracturability (1004.8 g·s), although these differences were not statistically significant. The use of defatted burdock seed (Xyl-DBS) produced the softest gingerbread cookies, with fracturability values comparable to those of the xylitol control (Xyl-C). Both native and defatted burdock seeds were ground using the same milling conditions prior to their incorporation into the formulations; therefore, a comparable particle size distribution can be expected. Although minor differences in particle size cannot be completely excluded, the observed changes in textural properties are more likely associated with compositional differences between native and defatted burdock seeds, particularly the reduced lipid content and the relatively higher proportion of protein and dietary fibre in the defatted material. These compositional differences may influence water binding capacity and the structure of the dough matrix, thereby affecting the texture of the final product.

A pronounced softening effect resulting from the replacement of sucrose with xylitol has been widely reported for various short-dough cookie types, including low-fat and home-made cookies [31,32,33,34]. Xylitol is more hygroscopic than sucrose and competes more effectively for water, thereby weakening the gluten network and reducing breaking strength [35]. Although xylitol also crystallizes during cooling, it forms fewer crystals than sucrose [33], resulting in a less rigid and less fragile cookie structure.

#### 3.2.4. Colour Properties

The investigated gingerbread cookies differed significantly in the instrumentally measured colour attributes of the upper crust (Table 8). Control gingerbread cookies prepared with sucrose (Suc-C) exhibited the darkest colour, characterized by the lowest lightness and the highest redness and yellowness values (L*, a*, and b*, respectively). Replacement of sucrose with xylitol resulted in a significant reduction in red and yellow colour components, accompanied by a slight increase in lightness of the upper crust. Lower colour attribute values in xylitol-sweetened cookies have also been reported [33].

It has been suggested that, unlike sucrose, xylitol does not participate in Maillard or caramelization reactions. However, Rutkowska et al. [34] demonstrated that xylitol can take part in Maillard reactions, although it leads to the formation of melanoidins with a lower browning index, likely due to its different molecular structure compared with sucrose. Incorporation of burdock seed, in both native and defatted forms, decreased lightness and significantly reduced redness and yellowness of the upper crust compared with the sucrose control, and also resulted in lower redness and yellowness compared with both control formulations (Table 8). In line with these findings, Lee et al. [15] reported a pronounced reduction in the L*, a*, and b* colour parameters of the cookie crust following supplementation with burdock seed powder. Burdock seeds are naturally dark, probably due to the presence of pigments and coloured phenolic compounds and lignans, which contribute to the darkening observed in the enriched products.

#### 3.2.5. Sensory Properties

Table 9 presents the sensory attributes and corresponding grades of the gingerbread cookie samples. In the evaluation of burdock seed–enriched gingerbread cookies, panellists identified aroma and bitter–pungent aftertaste as the most influential sensory attributes, followed by mastication behaviour and crumb appearance and structure at fracture. In contrast, overall appearance and upper surface characteristics were less affected by formulation differences.

All gingerbread samples achieved total sensory scores ranging from 18.33 points for the cookies enriched with defatted burdock seed to 19.65 points for the sucrose control (traditional gingerbread), classifying all samples within the “good” quality category. No statistically significant differences in total sensory scores were observed among the samples (*p* > 0.05).

Unexpectedly, no significant differences were detected in the bitter–pungent aftertaste among the samples, although burdock seed is known to impart bitterness. Several panellists described the sensation not as bitterness but rather as a clove-like pungent sharpness, which was also slightly perceived in samples without burdock seed. The highest intensity of bitter–pungent aftertaste, although not statistically significant, was perceived in the cookies containing defatted burdock seed. This may be attributed to a higher relative concentration of bitter compounds following fat removal; however, the overall effect on aftertaste intensity remained negligible. This suggests that the gingerbread spice mixture, particularly the presence of cloves, effectively masked the bitter notes. Cloves contain high levels of eugenol, a volatile phenolic compound that strongly influences aroma perception and may dominate the flavour profile of spiced bakery products [36,37]. Strong aromatic stimuli from spices can also modulate taste perception and reduce the perceived intensity of bitterness through cross-modal flavour interactions [38].

A decrease in aroma scores was observed in gingerbread samples formulated with xylitol, which is consistent with the role of sucrose as an effective aroma carrier. Sucrose plays a key role in aroma and flavour development and enhances aroma intensity [39]. Previous studies have reported that sucrose replacement in cookie and cake formulations is associated with reduced “buttery” and “caramel” aroma notes [40,41]. The absence of sucrose suppresses the formation of volatile compounds due to the limited availability of monosaccharides participating in thermal reactions during baking [39]. Moreover, sucrose replacement alters the food matrix structure, indirectly affecting aroma release and perception [39].

Xylitol incorporation resulted in a slightly drier and more crumbly crumb structure, reflected by a gradual decrease in scores for fracture appearance and crumb structure. Nevertheless, mastication scores were not adversely affected compared with the sucrose control, indicating acceptable textural performance of xylitol-containing formulations.

### 3.3. Storage Test

Gingerbread cookie samples were stored for 60 days under different conditions: in the dark at 40 °C and 4 °C, and at ambient temperature (25 °C) under light or dark exposure.

#### 3.3.1. Impact of Storage Conditions on Texture and Sensory Characteristics of Gingerbread Cookies

**Figure 1:**
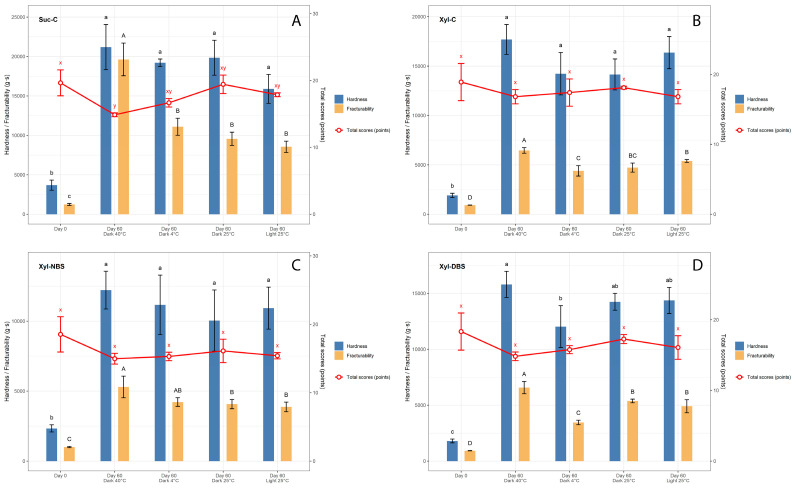
Changes in sensory and textural attributes of gingerbread cookie formulations during 60-day storage under different conditions (temperature and light). (**A**) control cookie with sucrose (Suc-C); (**B**) control cookie with xylitol (Xyl-C); (**C**) cookie with xylitol and 5% native burdock seeds (Xyl-NBS); (**D**) cookie with xylitol and 5% defatted burdock seeds (Xyl-DBS). Differences among groups were evaluated using one-way ANOVA followed by Tukey’s HSD post hoc test. Values marked with different letters indicate statistically significant differences at *p* < 0.05 (x, y for sensory scores; a, b, … for hardness; A, B, … for fracturability).

Changes in gingerbread cookie sensory and textural features are presented in Figure 1. A statistically significant reduction in sensory quality was detected only for the traditional sucrose-based gingerbread stored at 40 °C. For the remaining formulations, the greatest sensory deterioration was also observed at 40 °C; however, these differences did not reach statistical significance. Overall, only minor declines in sensory attributes were observed, and in most cases these changes were not statistically significant (Figure 1). Given the relatively low fat content of gingerbread cookies, storage at elevated temperatures did not result in a pronounced loss of sensory quality. Aroma intensity and crumb characteristics were the most affected attributes, with a noticeable weakening of aroma and increased crumb dryness. Storage at 4 °C led to a reduction in overall sensory quality, primarily due to adverse effects on crumb characteristics and mastication behaviour. In contrast, storage at 25 °C under dark conditions resulted in the smallest decline in sensory attributes.

Across all storage environments, both hardness and fracturability increased significantly over time (Figure 1). Among the tested formulations, sucrose-based gingerbread exhibited the most pronounced increase in fracturability when stored at 40 °C in the dark. Storage at 40 °C consistently resulted in the highest increases in hardness and fracturability across all samples, irrespective of formulation. In contrast, gingerbread prepared with xylitol showed markedly smaller changes in these textural parameters. The incorporation of burdock seed did not significantly affect texture evolution during storage, with the exception of hardness at 40 °C, where burdock-enriched gingerbread exhibited the smallest increase compared with the other formulations.

#### 3.3.2. Storage Stability of Individual Phenolics

The contents of the dominant phenolic compounds (arctiin, arctigenin, isochlorogenic acid, and chlorogenic acid) were quantified using high-performance liquid chromatography (HPLC). Analyses were performed on freshly prepared samples (day 0) and after 60 days of storage under various temperature and light conditions. The obtained results are presented in Table 10.

The results demonstrated that, irrespective of the applied storage conditions, no statistically significant changes were observed in the levels of the dominant phenolic compounds (*p* > 0.05). Although numerical variations of approximately 10–15% were observed for some compounds, these differences did not reach statistical significance due to variability among replicates (n = 3). These findings indicate a high degree of polyphenol stability in the formulated gingerbread cookies, supporting its suitability for long-term storage.

## 4. Conclusions

This study provides comprehensive evidence that burdock seed flour, particularly after supercritical CO_2_ defatting, is a valuable source of stable bioactive compounds and a functional ingredient suitable for bakery applications. The defatting process resulted in higher concentrations of key lignans, with arctiin remaining the predominant compound, alongside significant levels of phenolic acids such as isochlorogenic and chlorogenic acids. The stability of these phenolic compounds under various storage conditions highlights their resistance to thermal and photo-induced degradation, supporting the potential of burdock seed flour for use in processed food systems with extended shelf life. From a technological perspective, the incorporation of defatted burdock seed flour positively influenced dough and product texture, producing gingerbread cookies with reduced fracturability and softness comparable to the control formulation. These improvements can be attributed to the modified fibre composition and interactions between polysaccharides and the cookie matrix. In addition, the increased soluble dietary fibre content, particularly in xylitol-based formulations, further enhances the nutritional profile of the final products. Sensory evaluation confirmed that enrichment with both native and defatted burdock seed flour did not adversely affect consumer acceptability, as all samples were classified within the “good” quality category, with no significant differences among formulations. Storage studies indicated that sensory quality was mainly affected by elevated temperature, with significant deterioration observed only in sucrose-based cookies stored at 40 °C, whereas xylitol-containing and enriched formulations exhibited greater stability.

## Figures and Tables

**Table 1:** Working codes and descriptions of control and burdock-enriched gingerbread cookie formulations.

Code	Description
NBS	Native burdock seeds
DBS	Defatted burdock seeds
Suc-C	Control cookie with sucrose (0% burdock seeds)
Xyl-C	Control cookie with xylitol (0% burdock seeds)
Xyl -NBS	Cookie with xylitol and 5% native burdock seeds
Xyl -DBS	Cookie with xylitol and 5% defatted burdock seeds

**Table 2:** Ingredient list in gingerbread formulations.

Ingredients	Suc-C	Xyl-C	Xyl-NBS	Xyl-DBS
	[g]	[g]	[g]	[g]
Wholemeal spelt flour	70	70	70	70
Wholemeal rye flour	30	30	30	30
Sucrose	35	-	-	-
Xylitol	-	35	35	35
Honey	20	20	20	20
Vegetable fat	20	20	20	20
NBS	-	-	5	-
DBS	-	-	-	5
Cocoa	2	2	2	2
Spice mix ^1^	2	2	2	2
Lecithin	2	2	2	2
Sodium bicarbonate	1.5	1.5	1.5	1.5
Water	15	15	15	15

^1^ The spice mixture comprised cinnamon, nutmeg, coriander, ginger, fennel, cloves, allspice, anise, star anise, pepper, and cardamom.

**Table 3:** Sensory evaluation criteria for gingerbread cookies enriched with burdock seed.

Sensory Descriptors	Attribute Description	Scale Point Anchors (Min, Max)
Shape	Regularity	1 (deformed) → 5 (non-deformed, regular)
Upper surface	Appearance of cookie top surface, presence of cracks, colour	1 (smooth, burnt edges) → 5 (cracked, even, not burnt)
Fracture appearance	The way the cookie fractures when broken by hand, appearance of the fracture surface	1 (crumbly, jagged) → 5 (non-crumbly, smooth)
Crumb structure	Crumb structure development (pore size and distribution)	1 (dense, small pores, uneven) → 5 (well-developed pores, even)
Mastication	Sensations perceived during chewing and preparing for swallowing (food particle granulation, melting, disintegration, adhesion)	1 (hard and coarse crumb particles, slow particle disintegration and melting, adhesion to teeth) → 5 (excellent mastication properties, even disintegration of particles, fast and even melting, absence of coarse particles, absence of adhesion)
Aroma	Perception of the intensity of the gingerbread spice mix (aromatic combination of cinnamon, nutmeg, cloves, and ginger providing a complex flavour of strong, sweet, nutty, and savoury notes)	1 (absence) → 5 (highly pronounced)
Bitterly pungent aftertaste	Perception of the intensity of combined bitter notes similar to caffeine and peppery, warm, woody, and sharpness of cloves	1 (highly pronounced) → 5 (absence)

**Table 4:** Main lignans and phenolic compounds in native and defatted burdock seeds.

Sample	Arctiin ^a^	Arctigenin	Chlorogenic Acid	Isochlorogenic Acid
	[mg/g]	[mg/g]	[mg/g]	[mg/g]
NBS	68.30 ± 7.21	3.16 ± 0.52 a	2.29 ± 0.31	7.01 ± 0.66
DBS	75.16 ± 7.89	0.70 ± 0.01 b	2.63 ± 0.39	7.86 ± 0.82

^a^ Mean values (n = 3) followed by different letters within the same column differ significantly according to Tukey’s HSD post hoc test (*p* < 0.05); columns without letters indicate no statistically significant differences.

**Table 5:** Proximate composition, dietary fibre, carbohydrate composition, and energy value content of control and burdock-enriched gingerbread cookie formulations.

Formulation	Moisture ^a^	Ash	Proteins	Fat	Total Reducing Sugars	Total Dietary Fibres	Soluble Dietary Fibres	Insoluble Dietary Fibres	Total Carbohydrates	Available Carbohydrates	Energy Value	
	[%]	[%]	[g/100 g]	[g/100 g]	[g/100 g]	[g/100 g]	[g/100 g]	[g/100 g]	[g/100 g]	[g/100 g]	[kJ/100 g]	[kcal/100 g]
Suc-C	9.14 ± 0.21 b	1.41 ± 0.06	8.61 ± 0.25 b	11.90 ± 1.00	29.63 ± 2.11 a	6.75 ± 0.42	0.15 ± 0.02 b	6.60 ± 0.82	68.94 ± 3.48	62.19 ± 2.13	1813	431
Xyl-C	10.39 ± 0.24 a	1.39 ± 0.06	9.36 ± 0.27 a	12.18 ± 1.02	5.52 ± 0.39 b	6.16 ± 0.77	0.11 ± 0.01 b	6.05 ± 0.77	66.68 ± 3.05	60.52 ± 2.34	1793	426
Xyl-NBS	9.65 ± 0.23 ab	1.47 ± 0.07	8.70 ± 0.25 b	11.63 ± 0.98	3.60 ± 0.25 b	7.93 ± 0.92	0.11 ± 0.01 b	7.82 ± 0.98	68.55 ± 3.38	60.62 ± 1.98	1807	430
Xyl-DBS	8.98 ± 0.21 c	1.55 ± 0.07	7.56 ± 0.22 c	12.50 ± 1.05	6.49 ± 0.46 b	6.72 ± 0.83	0.29 ± 0.04 a	6.43 ± 0.80	69.41 ± 3.71	62.69 ± 2.38	1825	434

^a^ Mean values (n = 3) followed by different letters within the same column differ significantly according to Tukey’s HSD Post Hoc test (*p* < 0.05); columns without letters indicate no statistically significant differences.

**Table 6:** Mineral content and contribution to dietary reference intake (%DRI) of control and burdock-enriched gingerbread cookies.

Formulation	K ^a^	Ca ^b^	Mg	Fe	Zn	Cu	Mn
	[mg/100 g dw] (% DRI)	[mg/100 g dw] (% DRI)	[mg/100 g dw] (% DRI)	[mg/100 g dw] (% DRI)	[mg/100 g dw] (% DRI)	[mg/100 g dw] (% DRI)	[mg/100 g dw] (% DRI)
Suc-C	191.7 ± 16.1 (9.6)	27.2 ± 2.6 c (3.4)	60.3 ± 4.8 (16.1)	2.35 ± 2.22 (16.8)	1.48 ± 1.19 (14.8)	0.42 ± 0.02 (42.0)	1.34 ± 0.10 (67.0)
Xyl-C	189.6 ± 15.6 (9.5)	26.1 ± 2.0 c (3.3)	60.4 ± 4.7 (16.1)	2.31 ± 2.17 (16.5)	1.47 ± 1.20 (14.7)	0.40 ± 0.30 (40.0)	1.33 ± 1.16 (66.5)
Xyl-BS	194.1 ± 15.9 (9.7)	57.3 ± 4.4 b (7.2)	70.8 ± 5.5 (18.9)	3.02 ± 2.32 (21.6)	1.75 ± 1.35 (17.5)	0.44 ± 0.03 (44.0)	1.39 ± 0.12 (69.5)
Xyl-DBS	197.9 ± 16.2 (9.9)	63.7 ± 4.9 a (8.0)	69.8 ± 5.4 (18.6)	2.63 ± 0.20 (18.8)	1.67 ± 1.28 (16.7)	0.39 ± 0.30 (39.0)	1.38 ± 1.15 (69.0)

^a^ The percentage of dietary reference intake (%DRI) was calculated using the recommended daily DRI values for minerals (mg day^−1^): K, 2000; Ca, 800; Mg, 375; Fe, 14; Zn, 10; Cu, 1; and Mn, 2. ^b^ Mean values (n = 3) followed by different letters within the same column differ significantly according to Tukey’s HSD Post Hoc test (*p* < 0.05). Columns without letters indicate no statistically significant differences.

**Table 7:** Dimensional, volumetric, and textural properties of control and burdock-enriched gingerbread cookies.

Formulation	Spread ^a^	Specific Volume	Hardness	Fracturability
	[DH Ratio] ^b^	[g/mL]	[g × s]	[g × s]
Suc-C	3.56 ± 0.13 a	1.59 ± 0.04 a	3685.23 ± 644.38 a	1241.13 ± 124.67 a
Xyl-C	2.76 ± 0.22 bc	1.50 ± 0.02 b	1905.43 ± 219.05 b	921.22 ± 14.22 b
Xyl-NBS	2.58 ± 0.11 c	1.39 ± 0.03 c	2337.29 ± 256.70 b	1004.83 ± 42.32 b
Xyl-DBS	2.95 ± 0.11 b	1.48 ± 0.02 b	1803.38 ± 170.58 b	930.13 ± 14.17 b

^a^ Mean values (n = 3) followed by different letters within the same column differ significantly according to Tukey’s HSD Post Hoc test (*p* < 0.05). ^b^ DH ratio—Diameter to height ratio.

**Table 8:** Colour properties of the upper crust of control and burdock-enriched gingerbread cookies.

Formulation	L* ^a^	a*	b*
Suc-C	35.37 ± 0.67 a	15.03 ± 0.39 a	23.03 ± 0.40 a
Xyl-C	34.31 ± 1.66 ab	14.57 ± 0.20 a	20.62 ± 0.56 b
Xyl-NBS	32.81 ± 2.36 ab	13.08 ± 0.57 b	18.17 ± 1.25 c
Xyl-DBS	31.85 ± 1.73 b	13.09 ± 0.51 b	18.42 ± 1.02 c

^a^ L*—represents lightness on a scale from 0 (black) to 100 (white), a*—denotes the green (−) to red (+) axis, and b*—denotes the blue (−) to yellow (+) axis; Mean values followed by different letters within the same column differ significantly according to Tukey’s HSD Post Hoc test (*p* < 0.05).

**Table 9:** Sensory grades and quality classification of control and burdock-enriched gingerbread cookies.

Formulation	Appearance ^a^	Upper Surface	Fracture	Structure at Fracture	Mastication	Aroma	Bitterly Pungent Aftertaste	Total Score	Quality Class
	[Coef. 0.5]	[Coef. 0.5]	[Coef. 0.6]	[Coef. 0.6]	[Coef. 0.8]	[Coef. 1]	[Coef. 1]		
Suc-C	3.90 ± 0.76	3.60 ± 0.74	3.52 ± 0.73	3.95 ± 0.89	3.92 ± 0.61	4.30 ± 0.73	4.33 ± 0.82	19.65 ± 1.94	Good
Xyl-C	3.60 ± 0.63	3.87 ± 0.74	3.15 ± 0.89	3.45 ± 0.81	4.03 ± 0.88	3.97 ± 0.72	4.13 ± 0.92	18.90 ± 2.66	Good
Xyl-NBS	3.67 ± 0.84	3.87 ± 0.77	3.37 ± 0.77	3.33 ± 0.72	4.07 ± 0.78	3.80 ± 0.77	3.63 ± 1.08	18.53 ± 2.58	Good
Xyl-DBS	4.03 ± 0.61	3.59 ± 0.73	3.55 ± 1.10	3.73 ± 0.96	4.07 ± 0.88	3.87 ± 0.93	3.20 ± 1.08	18.33 ± 2.63	Good

^a^ No statistically significant differences between mean values were detected in any column according to ANOVA (*p* > 0.05); columns without letters indicate no statistically significant differences.

**Table 10:** Dominant lignans and phenolic compounds in burdock-enriched gingerbread cookies stored under different conditions.

Formulation	Storage Conditions	Arctiin ^a^	Arctigenin	Cholorogenic Acid	Isochlorogenic Acid
		[μg/g]	[μg/g]	[μg/g]	[μg/g]
Xyl-NBS	Day 0	1099.18 ± 67.52	572.58 ± 29.29	43.30 ± 5.62	460.35 ± 40.28
	Day 60 (25 °C, light)	922.72 ± 67.04	600.66 ± 65.87	39.97 ± 3.29	475.55 ± 24.39
	Day 60 (25 °C, dark)	966.75 ± 127.60	603.36 ± 86.73	40.97 ± 4.52	457.80 ± 66.46
	Day 60 (40 °C, dark)	937.99 ± 91.45	594.35 ± 51.12	40.78 ± 5.66	471.76 ± 29.78
	Day 60 (4 °C, dark)	977.30 ± 81.38	568.77 ± 65.87	42.42 ± 5.13	481.71 ± 44.89
Xyl-DBS	Day 0	1362.83 ± 155.99	199.94 ± 13.47	36.66 ± 4.07	458.83 ± 38.45
	Day 60 (25 °C, light)	1332.20 ± 136.62	201.17 ± 10.14	34.32 ± 3.06	444.64 ± 58.79
	Day 60 (25 °C, dark)	1390.62 ± 121.88	214.62 ± 31.43	36.90 ± 3.61	463.26 ± 51.47
	Day 60 (40 °C, dark)	1340.76 ± 145.85	194.35 ± 22.07	37.62 ± 4.97	467.31 ± 46.97
	Day 60 (4 °C, dark)	1304.88 ± 139.62	192.56 ± 18.33	36.95 ± 4.52	474.97 ± 35.95

^a^ No statistically significant differences were found among the mean values (n = 3) of the monitored compounds in relation to storage conditions.

## Data Availability

The original contributions presented in this study are included in the article/Supplementary Materials. Further inquiries can be directed to the corresponding author.

## Supplementary Materials

The following supporting information can be downloaded at: https://www.mdpi.com/article/10.3390/foods15071115/s1, Table S1: Validation parameters of the applied HPLC method.

## References

[1] Moro T.M.A., Clerici M.T.P.S. (2021). Burdock (*Arctium lappa* L) Roots as a Source of Inulin-Type Fructans and Other Bioactive Compounds: Current Knowledge and Future Perspectives for Food and Non-Food Applications. Food Res. Int..

[2] Shyam M., Sabina E.P. (2024). Harnessing the Power of Arctium Lappa Root: A Review of Its Pharmacological Properties and Therapeutic Applications. Nat. Prod. Bioprospecting.

[3] European Medicines Agency (2010). Community Herbal Monograph on *Arctium lappa* L., Radix. https://www.e-lactancia.org/media/papers/ArctiumBardanaBurdock-EMA2010.pdf.

[4] European Scientific Cooperative on Phytotherapy ESCOP Monograph (2016). Arctii Radix–Burdock Root. https://www.escop.com/downloads/arctii/.

[5] (2013). Physicians’ Desk Reference.

[6] (2015). Pharmacopoeia of the People’s Republic of China.

[7] (2016). Japanese Pharmacopoeia.

[8] Pljevljakušić D., Živković J., Petričević S., Aradski A.A., Radan M., Šavikin K., Ristić S. (2024). Dominant Lignan Profiles and Antidiabetic Activity of Thermally Treated and Non-Treated Burdock Seeds. Ind. Crops Prod..

[9] Liu J., Cai Y.-Z., Wong R.N.S., Lee C.K.-F., Tang S.C.W., Sze S.C.W., Tong Y., Zhang Y. (2012). Comparative Analysis of Caffeoylquinic Acids and Lignans in Roots and Seeds among Various Burdock (*Arctium lappa*) Genotypes with High Antioxidant Activity. J. Agric. Food Chem..

[10] Dziki D., Różyło R., Gawlik-Dziki U., Świeca M. (2014). Current Trends in the Enhancement of Antioxidant Activity of Wheat Bread by the Addition of Plant Materials Rich in Phenolic Compounds. Trends Food Sci. Technol..

[11] Cory H., Passarelli S., Szeto J., Tamez M., Mattei J. (2018). The Role of Polyphenols in Human Health and Food Systems: A Mini-Review. Front. Nutr..

[12] Kim J.H., Lee H.J., Lee H.-S., Lim E.-J., Imm J.-Y., Suh H.J. (2012). Physical and Sensory Characteristics of Fibre-Enriched Sponge Cakes Made with Opuntia Humifusa. LWT.

[13] Zucco F., Borsuk Y., Arntfield S.D. (2011). Physical and Nutritional Evaluation of Wheat Cookies Supplemented with Pulse Flours of Different Particle Sizes. LWT-Food Sci. Technol..

[14] Park B.R., Choi J.E., Lee J.H. (2017). Effect of Dried Hovenia Dulcis Fruit Powder on Quality Characteristics and Antioxidant Properties of Cookies. Korean J. Food Preserv..

[15] Lee J.H. (2017). Physicochemical and Sensory Evaluation of Wheat Cookies Supplemented with Burdock Powder. Food Sci. Preserv..

[16] Kim H.-Y., Kim K.-H., Yook H.-S. (2017). Quality Characteristics of Cookie with Burdock (*Arctium lappa* L.) Powder. Korean J. Food Cook. Sci..

[17] Wang D., Bădărau A.S., Swamy M.K., Shaw S., Maggi F., da Silva L.E., López V., Yeung A.W.K., Mocan A., Atanasov A.G. (2019). Arctium Species Secondary Metabolites Chemodiversity and Bioactivities. Front. Plant Sci..

[18] de Souza A.R.C., Guedes A.R., Rodriguez J.M.F., Bombardelli M.C.M., Corazza M.L. (2018). Extraction of Arctium Lappa Leaves Using Supercritical CO2 + Ethanol: Kinetics, Chemical Composition, and Bioactivity Assessments. J. Supercrit. Fluids.

[19] Ivanišová E., Mošaťová D., Hlaváčová Z., Hlaváč P., Kunecová D., Gálik B., Čech M., Harangozo Ľ., Kubiak P. (2023). Nutritional, Physical and Sensory Quality of Gingerbread Prepared Using Different Sweeteners. Agron. Res..

[20] Filipčev B., Šimurina O., Sakač M., Sedej I., Jovanov P., Pestorić M., Bodroža-Solarov M. (2011). Feasibility of Use of Buckwheat Flour as an Ingredient in Ginger Nut Biscuit Formulation. Food Chem..

[21] (2000). AOAC Official Methods of Analysis of AOAC International.

[22] Tańska M., Roszkowska B., Czaplicki S., Borowska E.J., Bojarska J., Dąbrowska A. (2016). Effect of Fruit Pomace Addition on Shortbread Cookies to Improve Their Physical and Nutritional Values. Plant Foods Hum. Nutr..

[23] Shi Y., Hu J., Wang H., Yan Z., Zhao G., Gao X., Li W., Qin K. (2022). Establishing a UHPLC-MS/MS Method for Evaluation of the Influence of Stir-Frying on the Pharmacokinetics of Seven Compounds in Arctii Fructus. RSC Adv..

[24] Liu Q.-D., Qin K.-M., Shen B.-J., Cai H., Cai B.-C. (2015). Optimization of the Processing Technology of Fructus Arctii by Response Surface Methodology. Chin. J. Nat. Med..

[25] (2010). EFSA Panel on Dietetic Products, Nutrition, and Allergies (NDA) Scientific Opinion on Dietary Reference Values for Carbohydrates and Dietary Fibre. EFSA J..

[26] European Parliament Council of the European Union Regulation (EC) No 1924/2006 of the European Parliament and of the Council of 20 December 2006 on Nutrition and Health Claims Made on Foods 2006. https://www.legislation.gov.uk/eur/2006/1924/contents.

[27] Republic of Serbia (2026). Regulation on Food Labelling, Presentation and Advertising. https://pravno-informacioni-sistem.rs/eli/rep/sgrs/ministarstva/pravilnik/2017/19/3/reg.

[28] Republic of Serbia (2025). Regulation on Nutrition and Health Claims Made on Food Labelling. https://pravno-informacioni-sistem.rs/eli/rep/sgrs/ministarstva/pravilnik/2018/51/2/reg.

[29] Kweon M., Slade L., Levine H., Martin R., Souza E. (2009). Exploration of Sugar Functionality in Sugar-Snap and Wire-Cut Cookie Baking: Implications for Potential Sucrose Replacement or Reduction. Cereal Chem..

[30] Lin S., Lee C., Mau J., Lin L., Chiou S. (2010). Effect of Erythritol on Quality Characteristics of Reduced-calorie Danish Cookies. J. Food Qual..

[31] Zoulias E.I., Piknis S., Oreopoulou V. (2000). Effect of Sugar Replacement by Polyols and Acesulfame-K on Properties of Low-Fat Cookies. J. Sci. Food Agric..

[32] Winkelhausen E., Jovanovic-Malinovska R., Velickova E., Kuzmanova S. (2007). Sensory and Microbiological Quality of a Baked Product Containing Xylitol as an Alternative Sweetener. Int. J. Food Prop..

[33] Mushtaq Z., Rehman S.-, Zahoor T., Jamil A. (2010). Impact of Xylitol Replacement on Physicochemical, Sensory and Microbial Quality of Cookies. Pak. J. Nutr..

[34] Rutkowska J., Baranowski D., Antoniewska-Krzeska A., Kostyra E. (2023). Comparison of Storage-Related Volatile Profiles and Sensory Properties of Cookies Containing Xylitol or Sucrose. Foods.

[35] Ma M., Han C.-W., Li M., Song X.-Q., Sun Q.-J., Zhu K.-X. (2019). Inhibiting Effect of Low-Molecular Weight Polyols on the Physico-Chemical and Structural Deteriorations of Gluten Protein during Storage of Fresh Noodles. Food Chem..

[36] Chaieb K., Hajlaoui H., Zmantar T., Kahla-Nakbi A.B., Rouabhia M., Mahdouani K., Bakhrouf A. (2007). The Chemical Composition and Biological Activity of Clove Essential Oil, *Eugenia Caryophyllata* ( *Syzigium Aromaticum* L. Myrtaceae): A Short Review. Phytother. Res..

[37] Czerny M., Schieberle P. (2002). Important Aroma Compounds in Freshly Ground Wholemeal and White Wheat FlourIdentification and Quantitative Changes during Sourdough Fermentation. J. Agric. Food Chem..

[38] Auvray M., Spence C. (2008). The Multisensory Perception of Flavor. Conscious. Cogn..

[39] Garvey E.C., O’Sullivan M.G., Kerry J.P., Kilcawley K.N. (2020). Factors Influencing the Sensory Perception of Reformulated Baked Confectionary Products. Crit. Rev. Food Sci. Nutr..

[40] Laguna L., Varela P., Salvador A., Fiszman S. (2013). A New Sensory Tool to Analyse the Oral Trajectory of Biscuits with Different Fat and Fibre Contents. Food Res. Int..

[41] Heenan S.P., Dufour J.-P., Hamid N., Harvey W., Delahunty C.M. (2010). The Influence of Ingredients and Time from Baking on Sensory Quality and Consumer Freshness Perceptions in a Baked Model Cake System. LWT-Food Sci. Technol..

