# Supplementary material for: Effects of Gingerbread Cookie Enrichment with Native and Supercritical CO2-Defatted Burdock Seeds
Source: Foods. 2026 Mar 24;15(7):1115. doi: 10.3390/foods15071115 (PMC13074170; doi:10.3390/foods15071115)
Supplement: Supplementary file 1 [file foods-15-01115-s001.zip › foods-4203648-supplementary.pdf]

Table S1 . Validation parameters of the applied HPLC method

Compound name	Regression equation	Linearity (R2)	Limit of detection (LOD) mg/mL	Limit of quantification (LOQ) mg/mL
Arctiin	y=4617.5x+15.374	0.9999	0.001	0.003
Arctigenin	y=9728.4x-73.467	0.9977	0.001	0.003
Chlorogenic acid	y=32329x-134.7	0.9999	0.002	0.006
Isochlorogenic acid	y=29896x-2716.8	0.9975	0.003	0.010
